# ProToxin, a Predictor of Protein Toxicity

**DOI:** 10.3390/toxins17100489

**Published:** 2025-10-01

**Authors:** Yang Yang, Haohan Zhang, Mauno Vihinen

**Affiliations:** 1 Computing Science and Artificial Intelligence College, Suzhou City University, Suzhou 215004, China; yyang@suda.edu.cn; 2 School of Computer Science and Technology, Soochow University, Suzhou 215008, China; 20235227013@stu.suda.edu.cn; 3 Suzhou Key Lab of Multi-Modal Data Fusion and Intelligent Healthcare, Suzhou 215004, China; 4 Department of Experimental Medical Science, Sölvegatan 19 B13, Lund University, SE-22 184 Lund, Sweden

**Keywords:** toxin, toxin prediction, machine learning, artificial intelligence, protein toxin

## Abstract

Toxins are naturally poisonous small compounds, peptides and proteins that are produced in all three kingdoms of life. Venoms are animal toxins and can contain even hundreds of different compounds. Numerous approaches have been used to detect toxins, including prediction methods. We developed a novel machine learning-based predictor for detecting protein toxins from their sequences. The gradient boosting method was trained on carefully selected training data. Initially, we tested 2614 features, which were reduced to 88 after a comprehensive feature selection procedure. Out of the four tested algorithms, XGBoost was chosen to train the final predictor. Comparison to available predictors indicated that ProToxin showed significant improvement compared to state-of-the-art predictors. On a blind test dataset, the accuracy was 0.906, the Matthews correlation coefficient was 0.796, and the overall performance measure was 0.796. ProToxin is a fast and efficient method and is freely available. It can be used for small and large numbers of sequences.

## 1. Introduction

Toxins are natural chemical compounds, peptides and proteins produced in all three kingdoms of life. They are poisonous substances to living organisms, sometimes including the toxin-producing organism itself. Venoms (zootoxins) contain up to hundreds of different compounds [1]. They are actively injected via bite or sting, or obtained, for example, by eating a venomous producer or through contact exposure. Venoms have multiple functions, often with many of them simultaneously, and are primarily contributed by different compounds and their combinations. The main function is to facilitate feeding. Another common purpose is defence. There are several other uses, usually limited to some organisms; see [1,2].

Venomous animals include snakes, spiders, scorpions, cone snails, centipedes, jellyfish, insects, sea anemones, lizards, fish, and platypuses. Many microbes and plants produce toxic compounds. Venoms have appeared in evolution at least 101 times [2]. The number of venomous animals is not known, but they appear in at least eight phyla [2].

Venoms pose a risk to humans. Venom effects are variable [3] and include cytotoxicity (causing cell and tissue damage, including necrosis), hemotoxicity (bleeding, blood clotting), and neurotoxicity (transmission of neural pulses), in addition to systemic effects, especially pain, swelling, in the most severe cases, respiratory failure, organ damage, and death. Effects can be mild and localised, severe or even life-threatening. 

It has been estimated that annually, approximately 100,000 people die from snake venom out of 2.7 million who are bitten [4]. Some 300,000 obtain disabling consequences. Other venomous organisms cause further cases. Some venom toxin derivatives are used as drugs [5,6] and there are several potential application areas; for a review, see Ref. [7]. Antivenom is a molecule that binds to the venom components, obstructing them, which prevents the venom from reaching its target cells, tissue or organ. Antivenom does not reverse the effects of venom; instead, it prevents further damage by filtering out unused toxins. Recently, artificial intelligence has started to be used in the design of antivenoms [8],

The toxicity of venom compounds has been widely studied, but details are known only for a limited number of compounds. Experimental methods provide the most reliable data, but since they are laborious, time-consuming, and expensive, computational approaches have gained popularity. Based on the existing knowledge, prediction methods have been developed to detect and identify toxin molecules. The tools can be classified into several categories. There are dedicated predictors for the toxicity of small compounds, including eToxPred [9], MolToxPred [10], ProTox-II [11], toxCSM [12], ToxMPNN [13], and ToxinPredictor [14].

Toxin-specific predictors include BTXpred for bacterial toxins [15], Deep-STP for snake toxins [16], DeTox [17] for venomous toxins, NTXpred for neurotoxins [18], and SpiderP to spider toxins [19].

Generic toxin predictors can be divided into peptide and protein toxin predictors. Amp-toxicity [20], ATSE [21], ClanTox [22], tAMPer [23], ToxGIN [24], ToxIBTL [25], and ToxinPred 3.0 [26] are for peptide toxins. CSM-Toxin [27], NNTox [28], ToxClassifier [29], ToxDL [30], TOXIFY [31], ToxinPred2 [32], and VISH-Pred [33] are for protein toxins.

We present a novel machine learning (ML)-based predictor for protein toxins. We collected an extensive feature set and applied it to a large set of toxin and non-toxin proteins obtained from UniProtKB. The gradient boosting algorithm demonstrated the best performance, outperforming existing tools in comparison.

## 2. Results

Although several methods are available for predicting protein toxicity, there is still room for performance improvements. We collected a large dataset of toxic and non-toxic proteins from UniProtKB [34]. Toxic proteins have been annotated in the database as such. The non-toxic proteins contain all types of proteins except those that are toxic or allergenic.

We used an innovative approach to select representatives for the non-toxic proteins. The sizes of the two datasets were so different that choosing cases among the non-toxic proteins was necessary. We used clustering-based sampling, which exposed the predictor to a more diverse range of negative samples representing various clusters in the feature space. First, we employed the elbow method to determine the optimal number of clusters (see Supplementary Figure S1) and then selected representatives from each cluster. This way, the negative set also included rare or less frequent sequences, which might have been overlooked if random sampling had been used. When the number of clusters was 8, the decline rate of the SSE curve was markedly reduced, indicating a transition from a steep drop to a more gradual flattening. We selected 8412 sequences from the 8 clusters. The method divided the sequences for non-toxic proteins into clusters based on their feature distributions.

**Figure 1:**
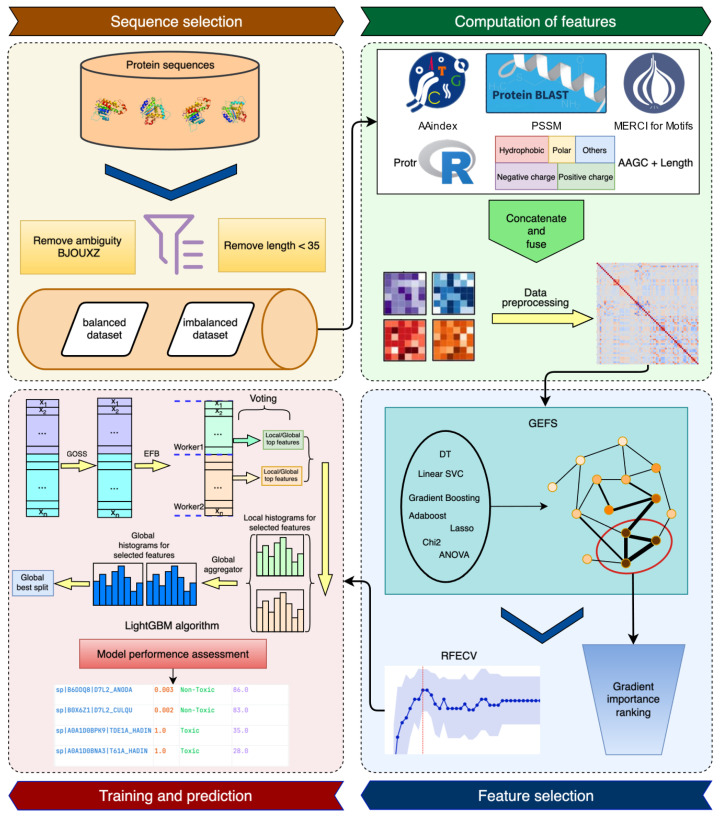
Flowchart of the ProToxin predictor development. After collecting the datasets, an extensive set of features was obtained. A multi-step procedure was then applied to reduce the number of features for training and testing the final predictor. The ProToxin predictor is freely available as a web service. GEFS, graphical ensemble feature selection.

Figure 1 shows the flowchart for the method development. A large number of features in six different areas were collected and used to describe the proteins. Feature selection was employed to identify a concise list of the most important features for training. Four algorithms were tested, and the best one was chosen to train the ProToxin method, which was then compared to the state-of-the-art methods.

### 2.1. The Choice of Algorithm

With the complete set of features, we trained four algorithms. We tested RF, SVM, and two versions of gradient boosting. All these algorithms have gained popularity in bioinformatics.

The results for the 20-fold CV are shown in Table 1. The gradient boosting predictors were clearly superior to the RF and SVM. A sensitivity of 0.871, PPV of 0.923, accuracy of 0.899, MCC of 0.800, OPM of 0.7281, and F1 of 0.896 were the best for XGBoost, but LightGBM was very close. The scores indicate an excellent performance.

We had a total of 16,824 proteins in the training set, which were described in 2614 dimensions. The number of toxin and non-toxin proteins was equal. All algorithms were run with default parameters. The XGBoost algorithm had the highest score among all metrics (Table 1). The SVM had the poorest performance and was clearly the slowest to run. RF was the fastest to train, but its performance was lower than that of the boosting methods.

### 2.2. Features and Feature Selection

We collected a large set of features (see Supplementary Table S1); therefore, feature selection was necessary to identify only those features that contribute to the prediction. Feature selection was performed in successive steps. We started by removing 16 features that had zero standard deviations. These features did not provide any information about the proteins. Then, all the features were compared to identify those that were correlated. For all feature pairs, we calculated the Pearson correlation. If the coefficient was ≥ 0.8, only one of the correlated features was retained. In this step, we deleted 1081 features, leaving us with 1517 features. LightGBM was used for feature selection since it is fast to learn. We employed feature selection with an ensemble method and obtained 100 features. The final set of features was obtained by applying RFECV to the top 100 features, yielding optimal performance with 88 features, as shown in Table 2.

Table 2 presents the results for various feature types and their combinations. There were only 20 PSSM features, one per amino acid. It showed a surprisingly good performance. Minimotifs were another feature with small numbers, 50. They were inferior in separating toxins from non-toxins. PPV and specificity were perfect for a method trained on minimotifs; however, all the other scores were the worst among the tested feature sets. Even amino acid groups and normalised sequence length with just seven features were substantially better. The best performance was achieved with 88 features from different categories. Specificity was somewhat better than sensitivity, 0.942 vs. 0.883. Still, the method can be considered quite balanced in performance. An accuracy and AUC of 0.912, and an MCC of 0.827 indicate a reliable predictor.

The selected features are listed in Table 3. The largest number of features was for protr-based composition/transition/distribution (CTD) descriptors, with 29 features, followed by dipeptide composition descriptors (21), conjoint triad descriptors (12), and AAindex and PSSM parameters (both 8). CTD features convert protein sequences into fixed-length numerical feature vectors based on their amino acid properties. Dipeptide composition features are for enrichment/depletion of dipeptides in sequences. To calculate the conjoint triad descriptors, first, amino acids are classified into seven groups based on their properties. Triads of these seven groups are used for 343-dimensional vectors that contain the frequencies of each continuous triad within a sequence.

**Figure 2:**
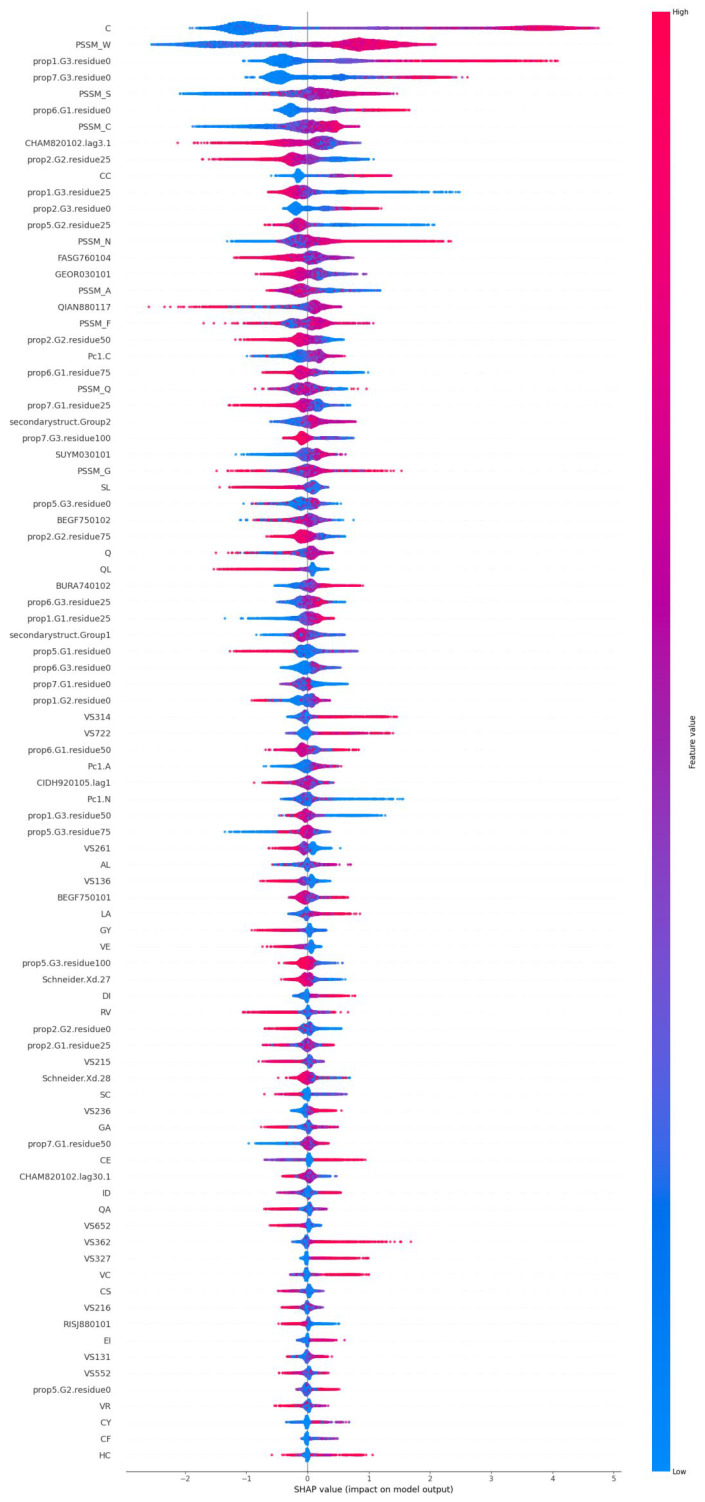
Shapley plot for the 88 selected features, organised in descending order of importance. The feature values are coloured based on their value, ranging from blue to red. The SHAP value indicates the impact of each feature on both positive and negative predictions. A positive result indicates a toxic protein, and a negative result indicates a non-toxic protein.

The importance of the features was visualised with the Shapley plot (Figure 2), which shows the importance of each feature in predicting toxic (positive values) and non-toxic (negative values) proteins. The features were arranged in order of decreasing importance. The blue colour indicates low values, and the red colour represents high values. The scale to the right indicates the increasing feature value.

The most important features are composition for C, sequence conservation PSSM scores for W, S and C. Among the selected features are two amino acid scores (for C and Q), 20 dipeptide scores, 8 PSSM values, and 8 AAindex values. Numerically, the largest group was the distribution descriptor. These features provide the position percentage in the entire sequence for the first residue, as well as 25%, 50%, 75%, and 100% residues for a certain encoded class. Among the top 10 most important features, four belong to this category. The physicochemical AAindex parameters describe various properties of amino acids. The significant parameters include those describing secondary structural elements (QIAN880117, BEGF750102, BURA740102, BEGF750101), linker propensities (GEOR030101, SUYM030101), pK values (FASG760104), and sequence conservation (RISJ880101).

Most of the features make the biggest contribution to toxin prediction, but there are, e.g., several frequency features for which small scores contribute to toxicity; see Figure 2. The Shapley plot summarises the importance of the selected features and provides information on how the scores are distributed among toxin and non-toxin proteins.

### 2.3. Method Development

To distinguish between LightGBM and XGBoost, which had very similar performances, we trained predictors using the selected 88 features and compared them on the blind test set, as shown in Table 4. The results are again very similar; however, XGBoost was chosen since it had a somewhat better overall performance. We call the final predictor ProToxin.

After selecting the best algorithm, we performed Bayesian hyperparameter optimisation of the XGBoost tool with cross-validation and trained the final predictor. The blind test set, an independent data set, was used for the final performance assessment. The accuracy was 0.906, the MCC was 0.797, and the OPM was 0.728. The scores for the blind test set are only marginally lower than those in feature selection; see Table 2.

### 2.4. Comparison to Other Methods

The blind test set was used to compare the performance of ProToxin to that of other available tools (Table 4). The comparison was made to five tools. For ToxinPred2, we tested two versions. ToxIBTL is a peptide toxin predictor; however, it was included in the comparison because it applies to proteins of up to 50 amino acids in length, and it was of interest to test whether it could extrapolate to proteins in general. Toxify and ToxIBTL are deep learning-based methods that utilise amino acid physicochemical propensities and evolutionary details. CSM-Toxin and VISH-Pred utilise language models: CSM-Toxin uses a bidirectional encoder representation from transformers (BERT), and VISH-Pred uses an ESM2 transformer model. ToxinPred2 is an RF model trained on many features, similar to ProToxin.

Apart from PPV and specificity, ProToxin performs markedly better than the competing tools. CSM-Toxin has the best scores for PPV and specificity, 0.976 and 0.989, respectively. However, this was achieved at the expense of severely biased performance. The scores for NPV and sensitivity are 0.861 and 0.728, respectively. A good predictor exhibits a well-balanced performance with minimal differences between the measures. The accuracy of CSM-Toxin was the second best after ProToxin. The MCC value was 0.774, and the OPM was 0.701. The ToxinPred2 hybrid version was the third-best method, followed by ToxinPred2.

MultiToxPred has the poorest performance with an accuracy of 0.598, MCC of 0.196, OPM of 0.201, and F1 of 0532. Interestingly, the peptide predictor ToxIBTL performed better despite being trained on shorter sequences; see Table 4.

### 2.5. ProToxin Server

ProToxin is available as an online platform. Users can manually enter or alternatively upload protein sequence(s) in FASTA format, upon which ProToxin retrieves and processes the essential sequence data. ProToxin is freely available at https://structure.bmc.lu.se/ProToxin/ (accessed on 26 September 2025) and https://www.yanglab-mi.org.cn/ProToxin/ (accessed on 26 September 2025). The datasets used for training are available at the websites.

## 3. Conclusions

We developed a novel gradient boosting-based predictor for protein toxicity. The method was trained on carefully selected training data. We employed a clustering approach to efficiently cover the space of non-toxic sequences. Initially, we tested a long list of various types of features, which were reduced to 88 after a comprehensive feature selection procedure. Out of the four tested algorithms, XGBoost was chosen to train the final predictor. Comparison to available predictors indicated that ProToxin showed significant improvement compared to state-of-the-art predictors. ProToxin is a fast and efficient method and is freely available. It can be used for small and large numbers of sequences. As experimental studies are expensive and time-consuming, ProToxin can provide a rapid assessment of toxicity. The tool could also be used for primary safety screening of sequencing-project-derived proteins and even designed proteins.

## 4. Materials and Methods

### 4.1. Data Collection and Cleaning

Data for venoms and toxins were collected from UniProtKB [34] (https://www.uniprot.org/) and downloaded on 9 September 2024. Sequences in FASTA format were retrieved using the keyword “KW-0800” for toxic proteins. Entries labelled as “reviewed” were selected. A reverse keyword-based search was employed to find a negative protein dataset. Sequences annotated with the keywords KW-0800 or KW-0020 (indicating allergenic proteins) were excluded. The sequences had a reviewed status. In both groups, sequences shorter than 35 amino acids were removed, and protein sequences containing ambiguous or nonstandard amino acids B, J, O, U, X, or Z were filtered out.

The training dataset contained 8412 toxins and 246,092 negative samples as non-toxins. An independent blind test set included 279 toxins and 474 non-toxins. Datasets are available at the predictor website.

There were many more non-toxic proteins than toxic ones. Therefore, we generated a balanced dataset. We applied the K-means clustering algorithm to the negative set. We clustered the sequences into 2 to 20 clusters (k values) and used the elbow method to evaluate the curve of the sum of squared errors (SSEs) as a function of k:

$$SSE=\sum_{i=1}^{n}\sum_{j=1}^{m}{\left\|x_{i}^{\left(j\right)}-c_{j}\right\|}^{2}$$

where $x_{i}^{\left(j\right)}$ denotes the sample that belonged to the *j* cluster; *cj* denotes the centre of the *j* cluster.

The balanced dataset was used to train the model. The positive and negative sequences were randomly divided into 20 subsets for cross-validation. In each split, 95% of the data was allocated for training, while the remaining 5% was reserved for validation. The performance evaluation of the cross-validation was calculated as the average result across 20 folds.

### 4.2. Features

We collected a large number of features belonging to six major groups, including protr-based features [35], PSSM scores, amino acid group counts [36], sequence length, AAindex features [37], and sequence motifs [38].

Protr program 1.7-4 [35], an R package v. 4.4.2, was used to generate most sequence-based features. With protr, we calculated features in 8 descriptor groups and obtained 1920 features altogether.

We used Psi-BLAST 12.14.0+ [39] to determine position-specific scoring matrices (PSSMs) based on evolutionary information. We searched for each protein against UniProtKB. The E-value threshold was set to 0.001, and the number of iterations was set to three. We used the global average pooling (GAP) method [7] to compress the irregularly shaped PSSMs into (1, 20).

Due to the varying lengths of each protein sequence, the shapes of the PSSMs differed. The matrix shape was (L, 20), where L is the number of amino acids in a protein. Using GAP, we mapped and concatenated the different matrices into the same shape (1, 20).

$${GAP}_{j}=\frac{1}{L}\sum_{i=1}^{L}P_{i,j}\;\forall j\in \{1,2,\ldots ,20\}$$

where L denotes the sequence length and *P_i,j_* represents the score of the j-th amino acid at position i in the sequence.

Amino acid group counts and sequence length. Amino acids were divided into six groups according to their physicochemical properties as follows: hydrophobic (V, I, L, F, M, W, Y, and C), negatively charged (D and E), positively charged (R, K and H), conformational (G and P), polar (N, Q and S) and others (A and T) [36]. We counted the numbers in the six groups and the sequence length. In this category, there were a total of seven features.

Sequence minimotifs were identified with Motif-EmeRging and with Classes-Identification (MERCI) [38]. We identified 50 motifs that appeared in the toxin proteins but not in the non-toxic ones. A one-hot method was used, creating a vector of 50 elements for each sample.

In total, we collected 2614 features for each protein.

### 4.3. Feature Selection

We used a two-step feature selection. First, we removed columns with a standard deviation of 0 for features where all samples had the same value. Then, we performed a pairwise Pearson correlation analysis on the remaining features using the following formula:

$$r=\frac{\sum_{i=1}^{n}\left({f_{p}}_{i}-\bar{f_{p}}\right)\left({f_{q}}_{i}-\bar{f_{q}}\right)}{\sqrt{\sum_{i=1}^{n}{\left({f_{p}}_{i}-\bar{f_{p}}\right)}^{2}}\sqrt{\sum_{i=1}^{n}{\left({f_{q}}_{i}-\bar{f_{q}}\right)}^{2}}},\forall p,q\left\{1,2,\ldots ,2614\right\},p\neq q$$

where *x_i_* and *y_i_* were the *i*th variables of features X and Y, respectively. $\bar{x}$ and $\bar{y}$ were the mean values for X and Y, respectively. If the correlation coefficient between the two features was ≥0.8, the two features exhibited a strong correlation, and one was removed.

Graphical ensembling theory [40] was used to identify the most important features among the remaining features with a co-selection graph, where vertices represented features and edges were weighted by the number of times the features were selected together, allowing for the training of several ML algorithms. We used recursive feature elimination with cross-validation (RFECV) [41] to obtain the final set from the top 100 features.

### 4.4. Model Architecture

We tested several ML algorithms, including random forests (RF), support vector machines (SVM), and two versions of gradient boosting methods: XGBoost and LightGBM. A 20-fold CV was used to identify the best-performing algorithm.

The ML algorithms utilised Python Scikit-learn 1.2.1 scripts and employed default parameters. We adopted a strict nested cross-validation approach and implemented an end-to-end, reproducible workflow using a Scikit-learn pipeline. This ensured that any step utilising label information was learned only with the training portion of each outer fold, and that transformations were applied only to the test portion of that fold, thereby preventing information leakage.

RF is an ensemble learning method based on decision trees [42]. It constructs multiple decision trees and aggregates their predictions through voting (for classification) or averaging (for regression). It is resistant to overfitting and robust to noisy data. It uses random feature selection and bootstrapped samples to construct multiple decision trees, improving generalisation. It can be computationally expensive with a large number of trees.

SVM is a supervised learning algorithm for classification and regression [43]. It finds the optimal hyperplane that maximises the margin between different classes. Kernel methods enable the effective handling of nonlinear classification problems. It also works well with small datasets and high-dimensional data.

XGBoost is an ensemble learning method based on gradient boosting that uses weighted decision trees for modelling [44]. It improves efficiency through optimisations like second-order derivative calculations of the loss function and regularisation techniques, balancing accuracy and computational performance.

LightGBM (Light Gradient Boosting Machine), developed by Microsoft in 2017, is optimized for large-scale data and high-dimensional features [45]. It utilizes GOSS (Gradient-based One-Side Sampling) to prioritize high-gradient samples and EFB (Exclusive Feature Bundling) to merge rarely co-occurring features, thereby boosting speed and reducing memory usage. It is much faster and lighter than XGBoost, and it supports parallel learning, GPU acceleration, and automatic handling of missing values.

### 4.5. Performance Assessment

We used 10 measures to describe the performance of models, including accuracy; area under the curve, AUC; F1 measure; negative predictive value, NPV; positive predictive value, PPV; Matthews correlation coefficient, MCC; overall performance measure, OPM; sensitivity; and specificity.

$$NPV=\frac{TN}{TN+FN}$$

$$PPV=\frac{TP}{TP+FP}$$

$$Specificity=\frac{TN}{TN+FP}$$

$$Sensitivity=\frac{TP}{TP+FN}$$

$$Accuracy=\frac{TP+TN}{TP+FP+TN+FN}$$

$$MCC=\frac{TP\cdot TN-FP\cdot FN}{\sqrt{\left(TP+FP)(TP+FN)(TN+FP)(TN+FN\right)}}$$

$$nMCC=\frac{1+MCC}{2}$$

$$OPM=\frac{\left(NPV+PPV)(Specificity+Sensitivity)(Accuracy+nMCC\right)}{8}$$

$$F1=\frac{2\cdot PPV\cdot Sensitivity}{PPV+Sensitivity}$$

AUC=Area under ROC curve

TP and TN are correctly predicted toxic and non-toxic cases, respectively, and FN and FP are the numbers of incorrect predictions for toxic and non-toxic cases, respectively.

## Figures and Tables

**Table 1:** Selection of the best algorithm by training on all features. Results are for 20-times CV. The best scores are in boldface.

	LightGBM	RF	SVM	XGBoost
TP	360.05	354.35	**368.4**	366.55
TN	389.95	**397.20**	329.55	389.75
FP	30.65	**23.40**	91.05	30.85
FN	60.55	66.25	152.20	**54.05**
NPV	0.866	0.857	0.684	**0.879**
PPV	0.922	**0.938**	0.747	0.923
Specificity	0.927	**0.944**	0.738	0.927
Sensitivity	0.856	0.842	0.638	**0.871**
Accuracy	0.892	0.894	0.711	**0.899**
MCC	0.786	0.791	0.426	**0.800**
OPM	0.711	0.717	0.363	**0.728**
F1	0.888	0.888	0.688	**0.896**
AUC	0.892	0.893	0.711	**0.899**

**Table 2:** Feature selection with LightGBM and 20-fold CV on the training dataset. The best scores are in boldface.

Features	Selected Features	Protr-Based Features	PSSM	AAindex	Amino Acid Groups + Length	Motifs
Dimension	88	1920	20	617	7	50
TP	**371.45**	357.45	369.10	357.90	300.65	31.10
TN	396.10	401.60	393.10	392.55	320.95	**420.55**
FP	24.50	19.00	27.50	28.05	99.65	**0.05**
FN	**49.15**	63.15	51.50	62.70	119.95	389.50
NPV	**0.890**	0.864	0.884	0.863	0.728	0.519
PPV	0.938	0.950	0.931	0.928	0.751	**0.999**
Specificity	0.942	0.955	0.935	0.933	0.763	**1.000**
Sensitivity	**0.883**	0.850	0.877	0.851	0.715	0.074
Accuracy	**0.912**	0.902	0.906	0.892	0.739	0.537
MCC	**0.827**	0.809	0.814	0.787	0.479	0.195
OPM	**0.761**	0.739	0.746	0.713	0.404	0.231
F1	**0.910**	0.897	0.903	0.887	0.732	0.137
AUC	**0.912**	0.902	0.906	0.892	0.739	0.537

**Table 3:** Selected features.

Feature Group	Feature Name	Feature Group	Feature Name
protr_ctd ^a^	prop2.G2.residue25	protr_ctriad	VS362
	prop1.G3.residue0		VS261
	prop7.G3.residue0		VS131
	prop7.G1.residue25		VS314
	prop5.G2.residue0		VS552
	prop1.G3.residue25		VS236
	prop5.G2.residue25		VS215
	prop6.G1.residue75		VS136
	prop5.G3.residue0		VS722
	prop6.G1.residue50		VS652
	prop5.G1.residue0		VS216
	prop2.G2.residue50		VS327
	prop2.G3.residue0	protr_dpc	SL
	prop1.G2.residue0		CS
	prop6.G1.residue0		QL
	prop2.G2.residue75		CC
	prop2.G1.residue25		VE
	secondarystruct.Group2		HC
	prop6.G3.residue25		CY
	prop7.G1.residue50		VC
	prop7.G3.residue100		RV
	prop7.G1.residue0		GY
	prop5.G3.residue100		EI
	prop1.G1.residue25		GA
	prop6.G3.residue0		LA
	prop5.G3.residue75		ID
	prop1.G3.residue50		VR
	secondarystruct.Group1		DI
	prop2.G2.residue0		AL
protr_mb	CHAM820102.lag3		QA
	CHAM820102.lag30		CF
protr_qso	Schneider.Xd.28		SC
	Schneider.Xd.27		CE
protr_apaac	Pc1.N	protr_geary	CIDH920105.lag1
	Pc1.A	protr_aac	C
	Pc1.C		Q
AAindex	FASG760104	PSSM	PSSM_W
	QIAN880117		PSSM_C
	GEOR030101		PSSM_Q
	RISJ880101		PSSM_S
	BEGF750102		PSSM_N
	BURA740102		PSSM_G
	BEGF750101		PSSM_F
	SUYM030101		PSSM_A

^a^ acc, amino acid composition; apaac, amphiphilic pseudo amino acid composition; ctd, composition/transition/distribution; ctriad, conjoined triad; dpc, dipeptide composition; geary, Geary autocorrelation; mb, normalized Moreau–Broto autocorrelation; paac, pseudo-amino acid composition; qso, quasi-sequence-order; PSSM; position-specific scoring matrix; AAaindex, AAindex descriptors.

**Table 4:** Comparison of the performance of toxin prediction methods on the blind test data. The best scores are bolded.

Model	TP	TN	FP	FN	NPV	PPV	Specificity	Sensitivity	Accuracy	MCC	OPM	F1	AUC	Coverage
ProToxin	225	457	17	54	0.894	0.930	0.964	0.806	**0.906**	**0.797**	**0.728**	0.864	0.885	1.000
XGBoost-88	229	453	21	50	**0.901**	0.916	0.956	**0.821**	**0.906**	0.796	**0.728**	**0.866**	**0.888**	1.000
ToxinPred2	194	451	23	85	0.841	0.894	0.951	0.695	0.857	0.690	0.608	0.782	0.823	1.000
ToxinPred2-hybrid	225	430	44	54	0.888	0.836	0.907	0.806	0.870	0.719	0.639	0.821	0.857	1.000
ToxIBTL	161	459	15	118	0.795	0.915	0.968	0.577	0.823	0.622	0.540	0.708	0.773	1.000
VISH-Pred	210	451	7	67	0.871	0.968	0.985	0.758	0.899	0.789	0.718	0.850	0.871	0.976
CSM-Toxin	203	469	5	76	0.861	**0.976**	**0.989**	0.728	0.892	0.774	0.701	0.834	0.859	1.000
MultiToxPred	172	278	196	107	0.722	0.467	0.586	0.616	0.598	0.196	0.214	0.532	0.601	1.000

## Data Availability

The datasets used to train and test the method are freely available at the ProToxin websites at https://structure.bmc.lu.se/protoxin/ (accessed on 26 September 2025) and https://www.yanglab-mi.org.cn/ProToxin/ (accessed on 25 September 2025).

## Supplementary Materials

The following supporting information can be downloaded at: https://www.mdpi.com/article/10.3390/toxins17100489/s1, Supplementary Figure S1: The elbow method was used to obtain the optimal number of clusters for the negative protein sequences, Supplementary Table S1: Distribution of the feature categories when having different numbers of features.
