# Supplementary material for: ProToxin, a Predictor of Protein Toxicity
Source: Toxins (Basel). 2025 Oct 1;17(10):489. doi: 10.3390/toxins17100489 (PMC12567798; doi:10.3390/toxins17100489)
Supplement: Supplementary file 1 [file toxins-17-00489-s001.zip › toxins-3849033-supplementary.pdf]

Supplementary Materials: ProToxin, a Predictor of Protein Toxicity

Yang Yang[1,2,3], Haohan Zhang[2] and Mauno Vihinen [4*]

[1] Computing Science and Artificial Intelligence College, Suzhou City University, Suzhou 215004, China; yyang@suda.edu.cn

[2] School of Computer Science and Technology, Soochow University, Suzhou 215008, China; 20235227013@stu.suda.edu.cn

[3] Suzhou Key Lab of Multi-Modal Data Fusion and Intelligent Healthcare, Suzhou 215004, China

[4] Department of Experimental Medical Science, Sölvegatan 19 B13, Lund University, SE-22 184 Lund, Sweden

* Correspondence: mauno.vihinen@med.lu.se

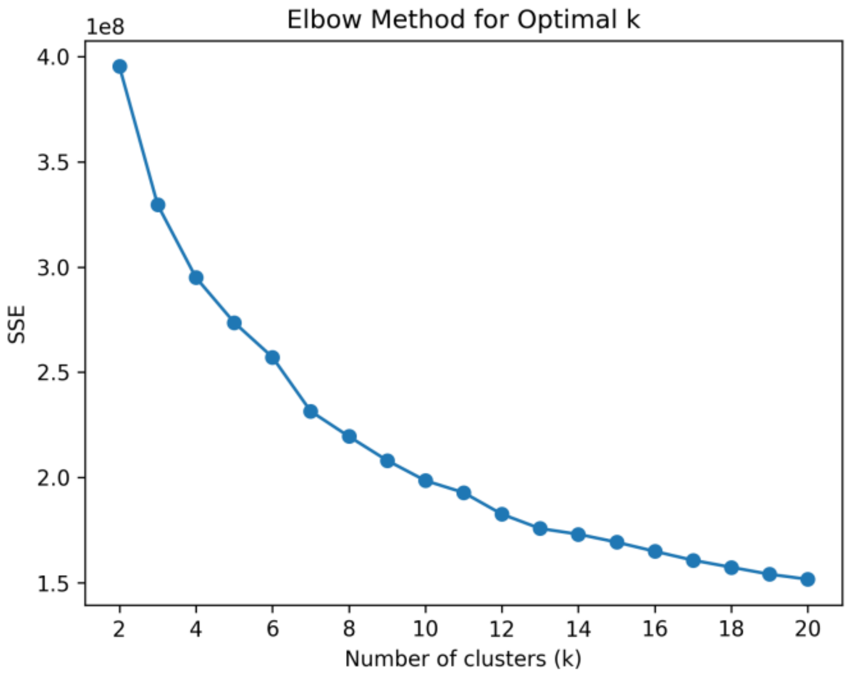

Supplementary Figure S1. The elbow method was used to obtain the optimal number of clusters for the negative protein sequences.

Supplementary Table S1. Distribution of the feature categories when having different numbers of features.

	Top 100	88 features	Top 50	Top 20
protr total	84	72	43	16
protr_aac[a]	2	2	2	1
protr_apaac	3	3	2	1
protr_ctd	29	29	19	12
protr_ctriad	19	12	6	
protr_dpc	26	21	12	2
protr_geary	1	1		
protr_mb	2	2		
protr_moran				
protr_paac				
protr_qso	2	2	2	
protr_socn				
pssm	8	8	5	3
aaindex	8	8	2	1

[a]acc, amino acid composition; apaac, amphiphilic pseudo amino acid composition; ctd, composition/transition, distribution; ctriad, conjoined triad; dpc, dipeptide composition; geary, Geary autocorrelation; mb, normalized Moreau-Broto autocorrelation; paac, pseudo-amino acid composition; qso, quasi-sequence-order; socn, sequence-order-coupling number; pssm; position-specific scoring matrix; aaindex, AAindex descriptors.
